# The role of exosomes in ankylosing spondylitis: from biological features and functional cargo to clinical applications

**DOI:** 10.3389/fmolb.2026.1744396

**Published:** 2026-02-16

**Authors:** Han-ying Yuan, Jia Xu, You-yu Zhang, Xuan Xi, Heng Pan, Shu-jing Zhao, Kai-xu Li, De-hong Li, Yan Lu

**Affiliations:** 1 School of Public Health, Gansu University of Chinese Medicine, Lanzhou, China; 2 Department of Clinical Laboratory, Gansu Provincial Hospital, Lanzhou, China; 3 Blood Transfusion Department, Gansu Provincial People’s Hospital, Lanzhou, China

**Keywords:** ankylosing spondylitis, biomarkers, exosomes, immune inflammation, microRNA

## Abstract

Exosomes are small extracellular vesicles secreted by nearly all cell types and widely distributed in body fluids. They not only mediate intercellular material transfer but also play an important role in the regulation of immune pathways. Given their diverse biological functions, studies investigating the regulatory roles of exosomes in ankylosing spondylitis (AS) are receiving increasing attention. The functions of exosomes in AS largely depend on their bioactive cargo, including microRNAs (miRNAs), circular RNAs (circRNAs), long non-coding RNAs (lncRNAs), proteins, and other molecules. In addition, exosome-induced intercellular communication and modulation of immune regulatory pathways are also critical. Recent studies have shown that exosomal crosstalk mechanisms may affect major AS-related pathways, such as immune responses, inflammatory signaling, and bone metabolism balance. This review summarizes the biological characteristics of exosomes and advances in their functional cargos in AS regulation. More animal and clinical studies are needed to explore the role of exosomes in AS. The ongoing development of sequencing technologies and biotechnology indicates that exosomes hold potential as diagnostic biomarkers for AS and provide new insights into its diagnosis and treatment.

## 1 Introduction

Ankylosing spondylitis (AS), also referred to as axial spondyloarthritis (Sieper and Poddubnyy, 2017), was a chronic inflammatory disease that primarily affected the sacroiliac joints and spine (Robinson et al., 2021). The global prevalence of AS has been estimated at 0.20%–0.25% among adults in North America and Europe; in China, the prevalence has been reported to be 0.29%, whereas in Arctic communities with the highest prevalence of human leukocyte antigen (HLA)-B27 worldwide, it has reached 0.35% (Navarro-Compán et al., 2021; Khan et al., 2023; Moro et al., 2025). AS predominantly affected young and middle-aged adults, severely compromising health and imposing substantial social and economic burdens (Crossfield et al., 2021).

The etiology of AS remained incompletely understood, and susceptibility was strongly associated with genetic, environmental, and lifestyle factors (Zhu et al., 2019), as well as with immune-related mechanisms involving multiple immune cells, mediators, and biomarkers (Pishgahi et al., 2020). Although the pathological mechanisms were not fully understood, HLA-B27 was consistently associated with AS prevalence across populations (Murphy et al., 2022; Li Z. et al., 2023). Beyond axial and peripheral joint involvement, clinical comorbidities including uveitis, ulcerative colitis, and psoriasis were commonly observed (Zhang et al., 2023). Diagnostic approaches included clinical assessment, such as inflammatory back pain and morning stiffness, together with imaging modalities including X-ray and MRI to detect sacroiliac inflammation or structural changes, and laboratory testing including HLA-B27 genotyping and measurement of inflammatory markers such as C-reactive protein and erythrocyte sedimentation rate (Wu et al., 2021; Agrawal et al., 2024).

However, diagnosis remained challenging due to atypical symptoms, subtle early imaging changes, and limitations of available biomarkers, which contributed to diagnostic delays and resulted in the loss of optimal treatment opportunities. With further elucidation of AS mechanisms, more effective diagnostic and therapeutic strategies were expected (Li H. et al., 2023).

**FIGURE 1:**
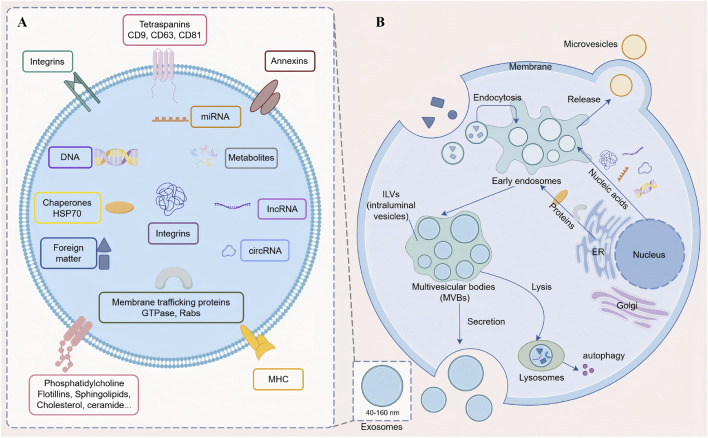
Structure and secretion mechanism of exosomes. **(A)** Schematic illustration of exosome structure and composition. Exosomes (40–160 nm) are membrane-bound extracellular vesicles with a phospholipid bilayer enclosing a hydrophilic lumen that carries diverse biomolecules (proteins, mRNAs, ncRNAs, DNA, and metabolites). **(B)** Overview of exosome biogenesis and secretion. Molecular cargo is internalized into early endosomes for sorting; recycled cargo returns to the plasma membrane or Golgi, while unrecycled cargo proceeds to late endosomes. Intraluminal vesicles (ILVs) form via endosomal membrane invagination, generating multivesicular bodies (MVBs) that either fuse with lysosomes for degradation or fuse with the plasma membrane to release ILVs into the extracellular space as exosomes.

Exosomes were nanoscale, membrane-bound extracellular vesicles (EVs) secreted by most eukaryotic cells, and carried a diverse array of biomolecules (Figure 1A), including proteins, messenger RNAs (mRNAs), non-coding RNAs (ncRNAs), DNA, and metabolites, which could be shuttled between cells (Jeppesen et al., 2019; Lee et al., 2019). Among these, ncRNAs—including microRNAs (miRNAs), circular RNAs (circRNAs), and long non-coding RNAs (lncRNAs)—were non-protein-coding RNAs that regulated gene expression, and accumulating evidence supported their involvement in the pathophysiology of inflammatory diseases (Kalluri and LeBleu, 2020; Saleem et al., 2024). Exosomes mediated the transfer of bioactive molecules between cells and played critical roles in intercellular communication (Raposo and Stahl, 2019). Accumulating evidence suggested that exosomes participated in AS pathogenesis through the regulation of inflammatory cytokines, immune responses, and bone metabolism. They acted not only as carriers of pathological signals such as miR-30c-5p and circ-0110634 but also as potential platforms for precision therapeutics, including mesenchymal stem cell (MSC)-derived exosomes and circRNA-targeted strategies, thereby attracting increasing attention in AS research. This review focuses on the specific roles of exosomes in the pathogenesis of AS, as well as research progress on their potential as biomarkers and therapeutic interventions.

## 2 Biological characteristics of exosomes

Exosomes were secreted by nearly all cell types, had a characteristic diameter ranging from 40 to 160 nm, and were abundantly present in bodily fluids such as urine, cerebrospinal fluid, and saliva. They performed essential functions, including the maintenance of cellular homeostasis, removal of cellular debris, and facilitation of intercellular and interorgan communication (Kalluri and LeBleu, 2020; Krylova and Feng, 2023). Exosomes were vesicular structures, with membranes composed of hydrophobic phospholipid bilayers and proteins that enclosed a hydrophilic lumen. Exosomal membranes usually contained membrane-associated functional proteins (Figure 1A), including diverse GTPases and Rab proteins (Rab11, Rab27a, Rab27b) involved in intracellular transport and fusion; integrins and tetraspanins (Alix, TSG101, CD9, CD63, CD81, and CD82) regulating cell adhesion; cytoskeletal proteins (actin and myosin); and heat shock proteins (HSP70, HSP90) (Milane et al., 2015; Blanc and Vidal, 2018). In addition, essential lipids of exosomal structure, such as phosphatidylcholine, phosphatidylserine, sphingolipids, and cholesterol, were also present on the membrane (Skotland et al., 2017).

Exosomes were first discovered by Pan and Johnstone in 1983 (Pan and Johnstone, 1983). With the advancement of research, extracellular vesicles were categorized into two major types based on their biogenesis and release mechanisms: microvesicles, which budded directly from the plasma membrane, and exosomes, which were released upon fusion of multivesicular bodies (MVBs) with the plasma membrane through exocytosis (Cocucci and Meldolesi, 2015; Zhang et al., 2015). This review focuses specifically on the latter—exosomes. Exosome biogenesis began with the internalization of molecular cargo into early endosomes, where initial sorting and fate determination occurred; some cargo was recycled to the plasma membrane or Golgi apparatus, whereas unrecycled cargo entered the endosomal maturation pathway. With changes in Rab proteins and membrane components, early endosomes progressively matured into late endosomes, during which invagination of the endosomal membrane led to the formation of intraluminal vesicles (ILVs) that encapsulated cargo, thereby giving rise to MVBs. MVBs could fuse with lysosomes for cargo degradation or could fuse with the plasma membrane to release ILVs into the extracellular space as exosomes (Figure 1B) (Wei et al., 2021).

The use of exosomes as carriers of biomarkers in the extracellular milieu has been well established; however, their clinical deployment remains constrained by the lack of standardization in exosome isolation and analytical workflows. Differential ultracentrifugation was often regarded as the gold standard for EV isolation; this approach effectively separated small EVs from large EVs based on size and density, thereby helping to reduce contamination (Zarovni et al., 2015). In parallel, size-based methods such as size-exclusion chromatography (SEC) separated exosomes from other EVs according to particle size and were expected to promote standardization in the field of exosome isolation, making clinical translation more feasible (Doyle and Wang, 2019). Immunoaffinity capture relied on antibodies to isolate exosomes according to the expression of surface antigens, enabling enrichment of exosomes from specific cellular sources and thereby mitigating contamination from non-exosomal EVs or lipoprotein/protein complexes in samples (Tauro et al., 2012). Meanwhile, EVs were classified into distinct categories according to size, density, biochemical composition, and biogenesis, and EV subtypes generated through different mechanisms typically fell within different size ranges. Because the size and physicochemical properties of different EV classes overlapped, it was difficult to isolate them in pure form, which in turn increased uncertainty in mechanistic attribution (Greening and Simpson, 2018). In addition, studies indicated that extracellular RNA could be encapsulated within EVs, and RNAs including miRNAs could be delivered in both EV-associated and non-EV-associated forms (Nik Mohamed Kamal and Shahidan, 2019). EV-encapsulated extracellular RNA has emerged as a key signaling modality and was commonly profiled using quantitative real-time PCR (qRT-PCR), microarrays, and RNA sequencing to characterize EV-associated RNAs, supporting their potential as biomarkers across diseases (Kumari et al., 2024). Therefore, in-depth dissection of noncoding and protein-coding RNAs within EVs will facilitate understanding of their functions and their potential utility as molecular markers.

## 3 Effects of exosomes/exosomal components on AS

Exosomes contained a variety of biological cargo, including nucleic acids, proteins, and other components such as lipids, chemical drugs, and natural substances (Zhang et al., 2019). Studies have demonstrated that exosomes played important roles in the development and progression of AS. Differential expression of exosomal cargo not only promotes or alleviates pathological processes but also provides potential targets for AS diagnosis and therapy.

### 3.1 Exosomal miRNAs in the pathogenesis of AS

Exosomal miRNAs have emerged as critical regulators in the pathogenesis of AS, influencing immune dysregulation, inflammation, and pathological osteogenesis (Table 1). Compared with healthy individuals, miRNAs in exosomes from patients with AS show heterogeneous expression patterns, with most miRNAs being upregulated and a minority being downregulated. Tavasolian et al. identified 24 differentially expressed miRNAs (22 upregulated and two downregulated), which regulated immune responses and chronic inflammation by modulating T cell functions (e.g., suppressing regulatory T cell proliferation, promoting T helper 17 cells (Th17) differentiation) and cytokine secretion (e.g., downregulating IL-8 and IL-10, upregulating IFN-α2 and IL-33) (Tavasolian et al., 2023). A systematic study reported 42 upregulated and 45 downregulated miRNAs in AS, identified miR-29 as the most frequently dysregulated miRNA (Li et al., 2021). Furthermore, exosomal miRNAs such as miR-125a, miR-451a (Fotoh et al., 2020), miR-146a, and miR-155 (Tan et al., 2022) exhibited promising diagnostic value and reflected disease activity and structural damage (Yildirim et al., 2021). Collectively, these findings suggest that exosomal miRNAs hold potential as noninvasive biomarkers and therapeutic targets for AS.

**TABLE 1:** Exosomal microRNAs in the Pathogenesis of AS.

Exosomal components	Expression pattern in AS	Origins	Effects and mechanisms	Source
miR-125a, miR-451a, miR-146a, miR-155	Differentially expressed	Patient-derived exosomes	Diagnostic potential; reflect disease activity and structural damage	Fotoh et al. (2020), Yildirim et al. (2021), Tan et al. (2022)
miR-30c-5p	Upregulated	Patient-derived exosomes	Targets IRF4; represses IRF4–Foxp3 synergy; inhibits Treg differentiation → loss of immune suppression, exacerbates AS immunopathology	Zheng et al. (2009), Mahnke et al. (2016), Ospelt (2024), Remalante-Rayco and Nakamura (2024)
miR-5189-3p	Suppressed by BMSC exosomes	BMSC-derived exosomes	Suppression activates BATF2/JAK2/STAT3 pathway, promotes FLS apoptosis, inhibits AS progression	Zhang et al. (2022b), Gerami et al. (2023)
miR-22-3p	Delivered via M2 macrophage EVs	M2 macrophage-derived EVs	Suppresses PER2 → relieves inhibition of Wnt7b → activates canonical Wnt/β-catenin pathway → enhances MSC osteogenic differentiation, exacerbates ectopic ossification	Liu et al. (2022)
miR-21	Upregulated (therapeutic)	AD-MSC-derived exosomes	Ameliorates spinal osteoporosis: ↑bone mineral density/content, ↓osteoclast activity (↓TRACP-5b, cathepsin K), ↓IL-6	Hu et al. (2022)
miR-92b-3p	Upregulated	Fibroblast-derived exosomes	Suppresses TOB1 → activates BMP/Smad pathway → promotes osteogenic differentiation and proliferation → pathological osteogenesis	Lu et al. (2023)

Sequencing analysis revealed that miR-30c-5p was significantly upregulated in exosomes from patients with AS compared with healthy controls and was among the most enriched exosomal miRNAs in AS. miR-30c-5p targeted interferon regulatory factor 4 (IRF4), a key factor in regulatory T-cell (Treg) differentiation (Ospelt, 2024). Through miRNA–gene targeting and gene–pathway interactions, miR-30c-5p suppressed IRF4 expression. IRF4 was a critical transcription factor for Tregs formation and function (Mahnke et al., 2016), and cooperated with forkhead box P3 (Foxp3) to maintain Treg functionality (Zheng et al., 2009). By downregulating IRF4 expression, miR-30c-5p disrupted the synergistic interaction between IRF4 and Foxp3, inhibited Treg differentiation, and consequently attenuated immune suppression, thereby exacerbating the immunopathology of AS (Remalante-Rayco and Nakamura, 2024).

Studies showed that mesenchymal stem cells (MSCs) from AS patients exhibited stronger osteogenic differentiation capacity than those from healthy donors, implicating AS-MSCs in pathological osteogenesis (Xie et al., 2016). Exosomes derived from bone marrow mesenchymal stem cells (BMSCs) played critical roles in modulating excessive inflammatory activation during skeletal sterile inflammation (Gerami et al., 2023). BMSC-derived exosomes suppressed miR-5189-3p, activated the BATF2/JAK2/STAT3 pathway, and promoted fibroblast-like synoviocyte (FLS) apoptosis, thereby inhibiting AS progression (Zhang Y. et al., 2022). EVs from M2 macrophages acted as carriers of miR-22-3p and transferred it into MSCs. Within recipient cells, miR-22-3p suppressed period circadian regulator 2 (PER2), thereby relieving inhibition of Wnt7b, activating the canonical Wnt/β-catenin signaling pathway, and enhancing MSC osteogenic differentiation, which exacerbated ectopic ossification in AS (Liu et al., 2022). Conversely, exosomal miR-21 derived from adipose-derived MSCs (AD-MSCs) showed therapeutic potential. Injection of miR-21-enriched exosomes into AS model mice ameliorated spinal osteoporosis by increasing bone mineral density and content, inhibiting osteoclast activity (reducing TRACP-5b and cathepsin K levels), and downregulating the proinflammatory cytokine IL-6 (Hu et al., 2022). In fibroblasts, upregulated miR-92b-3p directly suppressed TOB1 expression, thereby activating the BMP/Smad signaling pathway, which drove osteogenic differentiation and cell proliferation, contributed to pathological osteogenesis in AS (Lu et al., 2023).

### 3.2 Exosomal circRNAs in AS pathogenesis

CircRNAs were a class of noncoding RNAs with covalently closed-loop structures resistant to exonuclease digestion (Barrett and Salzman, 2016). With advances in RNA sequencing and bioinformatic prediction, numerous circRNAs had been identified as regulators of development, localization, and tissue-specific expression (Chen, 2016). In AS patients, exosomal circRNAs such as *hsa_circ_0110797*, *hsa_circ_0097378*, *hsa_circ_0122309*, *hsa_circ_0058275*, and *hsa_circ_0008346* were downregulated, and the differentially expressed circRNAs were mainly associated with negative regulation of nuclear factor kappa-light-chain-enhancer of activated B cells (NF-κB) activity and bone remodeling, processes implicated in AS (Zhang L. et al., 2022). Construction of circRNA–miRNA–mRNA interaction networks provided new insights into circRNAs as potential biomarkers via regulation of miRNAs and their target genes. CircRNAs could act as “sponges” for miRNAs, sequestering and modulating their activity, thereby regulating gene expression in various diseases (Xiao et al., 2022). A cross-analysis of AS patient platelets and spinal ligament tissues identified two downregulated circRNAs (circPTPN22 and circFCHSD2), whose target mRNAs were enriched in pathways such as Th17 cell differentiation, inflammatory bowel disease, cell adhesion molecules, cytokine–cytokine receptor interactions, Jak–STAT, and Wnt signaling, all involved in bone remodeling and immune regulation in AS (Wang et al., 2021).

Exosomal circRNAs, as emerging noncoding RNAs mediating intercellular communication, precisely regulated inflammation and bone metabolism balance in AS. hsa_circ_0003307 was significantly upregulated in peripheral blood mononuclear cells (PBMCs) of AS patients; knockdown of *hsa_circ_0003307* suppresses phosphoinositide 3-kinase (PI3K)/protein kinase B (AKT) pathway activation and reduced expression of key inflammatory mediators TNF-α and TNFAIP2, thereby alleviating synovial cell inflammation (Fang et al., 2022). Exosomal circ-0110634 derived from AS-MSCs was significantly elevated compared with healthy donors. After being delivered into recipient cells, circ-0110634 simultaneously bound TNF receptor-associated factor 2 (TRAF2) and TNFRII, promoted TRAF2 dimerization and ubiquitination-dependent degradation, disrupted the TRAF2–TNFRII interaction, and ultimately inhibited NF-κB and Mitogen-activated protein kinase (MAPK) signaling, two critical pathways driving osteoclast differentiation, thereby suppressing osteoclastogenesis (Ji et al., 2022).

### 3.3 Differential expression of exosomal lncRNAs in the pathogenesis of AS

LncRNAs were key epigenetic regulators that played essential roles in the pathogenesis of AS, as well as in the assessment of disease activity and therapeutic response (Sun et al., 2022). Multiple studies using patient serum, cartilage tissue, and synovial cells revealed a complex regulatory network of lncRNAs.

Various lncRNAs acted as competing endogenous RNAs (ceRNAs) to sequester miRNAs, thereby fine-tuning the expression of downstream target genes and signaling pathways, ultimately influencing chondrocyte fate and inflammatory states (Xie et al., 2020). It was reported that highly upregulated in liver cancer (HULC) was significantly upregulated in AS cartilage tissue; through a “sponging” effect it suppressed miR-556-5p expression, thus releasing miR-556-5p–mediated inhibition of the proto-oncogene yes-associated protein 1 (YAP1), ultimately exacerbating chondrocyte inflammation and suppressing proliferation (Yi et al., 2023). Similarly, metastasis-associated lung adenocarcinoma transcript 1 (MALAT1) was elevated in AS, where it sponged miR-558 to relieve inhibition of the pyroptosis effector GSDMD, thereby promoting chondrocyte pyroptosis and inflammation, while MALAT1 knockdown effectively attenuated this pathological process (Chen et al., 2022). In addition, the therapeutic effect of triptolide (TPL) was shown to be closely associated with regulation of the lncRNA NONHSAT227927.1. This lncRNA was highly expressed in AS and was a risk factor for disease activity; TPL exerted its anti-inflammatory effect by suppressing NONHSAT227927.1 expression, thereby inhibiting activation of the JAK2/STAT3 signaling pathway (Ding et al., 2024). High-throughput sequencing studies expanded the lncRNA landscape, identifying 145 differentially expressed lncRNAs (72 upregulated and 73 downregulated) in the serum of AS patients. Functional enrichment analysis revealed that these lncRNAs were mainly involved in immune-inflammatory processes such as protein ubiquitination, MHC class I antigen presentation, MAPK activation, and the IL-17 signaling pathway, and ceRNA network construction further demonstrated their complex regulatory roles in AS (Kou et al., 2024).

### 3.4 Differential expression of exosomal proteins and their relationship with AS progression

In recent years, proteomic studies of serum-derived EVs from AS patients have revealed significant alterations in their protein composition. It was found that the protein profile of serum-derived EVs in AS patients differed markedly from that of healthy controls. One study identified 73 differentially expressed proteins by LC-MS/MS, including 31 upregulated and 42 downregulated, which were significantly enriched in pathways such as “complement and coagulation cascades,” “*Staphylococcus aureus* infection,” “systemic lupus erythematosus,” and the “PI3K-Akt signaling pathway,” indicating that protein cargos carried by EVs might contribute to AS pathogenesis by modulating these key biological processes (Huang et al., 2020). Notably, a combination of LC-MS/MS and enzyme-linked immunosorbent assay (ELISA) identified a panel of protein biomarkers specifically upregulated in serum EVs from AS patients. Among them, serum amyloid A-1 (SAA1) was confirmed to be significantly overexpressed, suggesting strong potential as a diagnostic biomarker for AS. In addition, Fibulin-1, von Willebrand factor, complement factor H-related protein 2, and lysozyme C were also consistently found to be elevated in AS serum EVs (Sung et al., 2025).

In serum-derived exosomes from AS patients, proteins show increased expression during AS progression and were implicated in inflammatory responses. For example, follistatin-like protein one and IL-17A were elevated in patient exosomes and exhibited certain discriminatory ability compared with healthy controls. Compared with plasma, exosomes contained more intracellular and transmembrane proteins involved in critical biological processes (Colombo et al., 2014). In AS, processes such as inflammatory responses, responses to bacteria, cellular responses to growth factor stimulation, and the PI3K/Akt signaling pathway were particularly enriched in small exosomes. Moreover, macroautophagy and positive regulation of NF-κB transcriptional activity were enriched in large exosomes, whereas small exosomes showed high expression of follistatin-like protein 1 (Wu et al., 2023). Follistatin-like protein one was a pro-inflammatory molecule that promoted the production of pro-inflammatory cytokines (Cheng et al., 2017). For instance, following *Streptococcus* pneumoniae infection, follistatin-like 1 (FSTL1) positively regulated the nucleotide-binding oligomerization domain-containing protein (NOD)-like receptor family pyrin domain-containing 3 (NLRP3) inflammasome and promoted inflammatory injury via the TLR4/NF-κB signaling pathway (Chen and Liu, 2019). FSTL1 was therefore likely to be closely associated with the infectious etiology and inflammatory regulation of AS and might represent a potential pathological mechanism and therapeutic target.

Exosomal IL-17A could activate the JAK-STAT3 signaling pathway, thereby inducing the expression of matrix metalloproteinase-14 (MMP14) in the ligaments of patients with AS (Wang et al., 2024). MMP14 was involved in normal physiological processes such as tissue development, reproduction, and tissue remodeling, while also contributing to pathological processes such as arthritis and tumor metastasis (Conlon and Murray, 2019). Overexpression of MMP14 could lead to alterations in cytoskeletal and mechanotransduction pathways in MSCs and other cells, potentially driving pathological new bone formation (Kasper et al., 2007). Inhibition of IL-17A activity and exosome endocytosis could effectively suppress inflammation and pathological osteogenesis, thereby controlling AS progression.

In recent years, multiple exosomal miRNAs, circRNAs, lncRNAs, and proteins were investigated as potential diagnostic biomarkers for AS (Fang and Liu, 2023); however, these molecular classes differ in their relative diagnostic value and clinical applicability. In many studies, exosomal miRNAs demonstrated superior diagnostic performance; for example, miRNAs such as miR-21 were associated with inflammatory markers including TNF-α and aberrant osteogenesis in patients with AS, indicating substantial potential as early, non-invasive indicators of disease activity (Zou et al., 2020; Sekar, 2021). Numerous lncRNAs involved in the regulation of inflammatory signaling, including H19, MEG3, and LOC645166, were dysregulated in AS, suggesting that lncRNAs might serve as novel biomarkers for diagnosis and prognostication of AS outcomes (Sun et al., 2022). By contrast, exosomal circRNAs represented an emerging area of research; although differential expression of certain circRNAs was identified in patients with AS and they might serve as biomarkers, their diagnostic accuracy metrics were not yet adequately validated (Zhang L. et al., 2022). Finally, exosomal proteins, such as surface markers and inflammation-related proteins, showed differential expression in patients with AS and could complement RNA-based biomarkers; however, because their expression might be influenced by a broader range of systemic diseases, their specificity for AS diagnosis could be lower (Huang et al., 2020). Overall, current evidence suggests that miRNAs and lncRNAs currently show strong diagnostic potential when used individually, while circRNAs and protein biomarkers hold promise as part of a combination of biomarkers to improve clinical diagnostic efficiency.

## 4 Exosomes/Exosomal components with potential impact on AS progression

**FIGURE 2:**
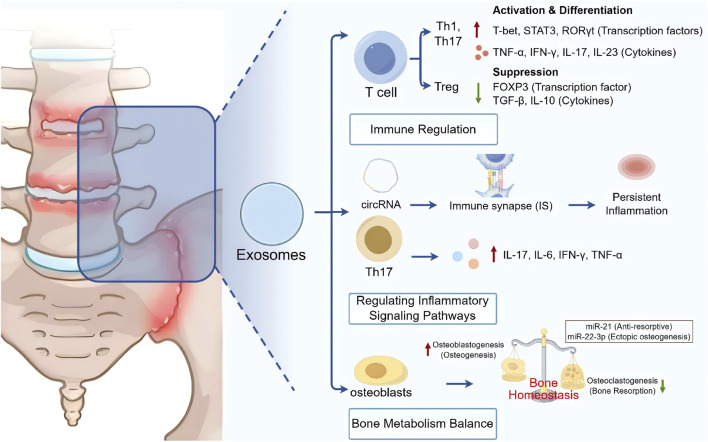
Exosomes participate in immune regulation, pro-inflammatory pathways, and the balance between osteogenesis and osteoclastogenesis to intervene in the pathological process of AS.

Ankylosing spondylitis progressed through distinct stages of immune imbalance and chronic inflammation, bone destruction, and pathological osteogenesis (Tam et al., 2010). Exosomes actively participated in these processes by modulating immune cell dysfunction, regulating T cell survival signaling, activating pro-inflammatory pathways, and interfering with the balance between osteogenesis and osteoclastogenesis, thereby exerting significant influence on AS pathology (Figure 2).

### 4.1 Exosome-mediated immune regulation

During AS progression, the cytokine profile and expression of T cells can be regulated by exosomal proteins. One study reported that exosomes derived from AS patient PBMCs altered T cell profiles and induced normal T cells into an inflammatory state by upregulating transcription factors (RORγt, STAT3, and T-bet) and cytokines (IL-17, IL-23, TNF-α, and IFN-γ) in Th1 and Th17 cells, while downregulating Treg cytokines (TGF-β and IL-10) and transcription factors (FOXP3) (Jafarpour et al., 2022).

Multiple studies demonstrated that different miRNAs were expressed in both innate and adaptive immune cells and played crucial roles in their development and function, including regulation of inflammation and T cell responses in AS (Tavasolian et al., 2022). The immunoregulatory roles of exosomes included antigen presentation, activation and suppression of immune responses, and expression of opsonins and complement factors (Greening et al., 2015). Notably, activated T cells could release miRNA-enriched exosomes at the immune synapse in a targeted manner; these exosomes acted as key information carriers, transferring regulatory RNA molecules to antigen-presenting cells (APCs) or other immune cells, thereby amplifying or maintaining aberrant immune activation states, ultimately leading to chronic inflammation and pathological new bone formation.

### 4.2 Exosomes participate in AS by regulating inflammatory signaling pathways

The expression of proteins and miRNAs in plasma exosomes of AS patients differs from that of healthy controls, suggesting that abnormally expressed exosomal miRNAs and proteins may intervene in the progression of AS. miRNAs functioned as a group of gene regulators and might originate from intracellularly modified expression or extracellular circulation. These circulating miRNAs could be transferred into the immune synapse (IS) via exosomes, transmitting signals to recipient cells and thereby initiating inflammatory signaling pathways in AS (Tavasolian et al., 2020; Bauer et al., 2022; van Niel et al., 2022). Exosomes from AS patients could induce significant upregulation of interferon-α2 and interleukin-33 in healthy CD4^+^ T cells, while suppressing the proliferation of Tregs, indicating that exosomes in AS activated T cells to further drive inflammation (Tavasolian et al., 2023). Th17 cells, a novel CD4^+^ T-cell subset, were characterized by the production of proinflammatory cytokines including IL-17, IL-6, IL-22, IFN-γ, and TNF-α (Pishgahi et al., 2020), and exerted negatively regulatory effects on immune responses (Liao and Tsai, 2023).

### 4.3 Exosomes intervene in bone metabolism balance and intercellular communication in AS

Exosomes influenced bone metabolic balance by modulating inflammatory responses, indirectly affecting the activities of osteoblasts and osteoclasts, thereby disrupting bone homeostasis (Longevity, 2024). Exosomes could transfer circRNAs (such as circPTPN22 and circFCHSD2) and regulate signaling pathways including JAK/STAT and NF-κB, thereby influencing fibroblast-like synoviocyte apoptosis and bone remodeling. Studies have shown that exosomal miR-21 induces the proliferation and differentiation of MSCs, increases bone mineral content and bone mineral density, and ameliorates osteoporosis symptoms in AS patients; in AS mouse models, it significantly reduced the number of osteoclasts, thereby decreasing bone resorption. In addition, it reduced IL-6 secretion, promoted IL-10 expression, and attenuated inflammation, thereby alleviating AS-related osteoporosis (Hu et al., 2022).

Expression of miR-22-3p in macrophage-derived exosomes was increased and positively correlated with spinal syndesmophyte formation and ectopic bone formation in AS. In AS mouse models, exosomal miR-22-3p from M2 macrophages was overexpressed and transferred into bone marrow MSCs, where it suppressed the expression of circadian regulator PER2, thereby affecting downstream pathways (Liu et al., 2022). This process simultaneously promoted the expression of runt-related transcription factor 2 (RUNX2) and osteocalcin. MSC-derived exosomes, through their bioactive cargos, exhibited considerable therapeutic potential in regulating bone metabolism balance in axial spondyloarthritis (AxSpA) (Tavasolian and Inman, 2023). These vesicles originated from MSCs, with core cargos including diverse miRNAs, proteins, and signaling molecules, which together constituted their “cargo” (Liu et al., 2024). Mechanistically, MSC-EVs influence disease progression through immunological regulation and tissue remodeling. They can deliver immunosuppressive or anti-inflammatory miRNAs to inflamed joints, suppressing overactivated immune cells and thereby alleviating local inflammation. Simultaneously, these vesicles can deliver signals promoting tissue repair and regeneration, regulate the activity balance between osteoblasts and osteoclasts, and are expected to suppress pathological new bone formation, such as spinal ankylosis, while promoting normal bone homeostasis.

## 5 Exosomes as potential diagnostic and therapeutic targets for AS

Disease-associated exosomes have been detected in human body fluids. Advances in sequencing technologies and biotechnology indicate that exosomes hold potential as diagnostic biomarkers for AS, offering new approaches for diagnosis.

Significant progress has been made in circulating miRNA studies for diagnosis and biomarker discovery. Evidence indicated that miR-138-5p expression was markedly decreased in the peripheral blood of AS patients, while TGF-β3 levels were elevated, and both were significantly correlated with disease activity indicators (HLA-B27, erythrocyte sedimentation rate (ESR), C-reactive protein (CRP), Bath Ankylosing Spondylitis Disease Activity Index (BASDAI)). Notably, the combined detection of these two markers had demonstrated strong diagnostic performance for AS (Cheng and Zhang, 2025). Furthermore, serum miR-3620-3p was identified as a highly promising AS-specific diagnostic biomarker. Its expression was significantly lower in AS patients compared with RA patients and healthy controls, showing high sensitivity and specificity, and its expression level was associated with the occurrence of uveitis as an AS complication (Lee et al., 2022). In clinical diagnosis, circRNAs also exhibited great potential. It was found that *hsa_circ_0079787* expression in the peripheral blood of AxSpA patients was significantly lower than that in healthy controls and systemic lupus erythematosus (SLE) patients, effectively distinguishing AxSpA from SLE, suggesting its potential as a novel, highly specific biomarker (Luo et al., 2020). Multiple lncRNAs also demonstrated excellent biomarker potential. Maternally expressed gene 3 (MEG3) and taurine upregulated gene 1 (TUG1) were significantly downregulated in the serum and tissues of AS patients. Their expression levels not only effectively differentiated patients from healthy controls but also negatively correlated with disease activity (BASDAI score) and disease duration. Patients with lower MEG3 or TUG1 expression tended to have longer hospital stays and higher readmission rates, suggesting their value in assessing disease severity and prognosis (Liu et al., 2019).

Exosomes also demonstrate significant value in AS therapy and in reflecting therapeutic effects. A comparative analysis before and after 3 months of anti-TNF therapy revealed that the expression of miR-130a-3p, miR-146a-5p, miR-21-5p, miR-22-3p, miR-23a-3p, miR-30a-5p, miR-362-3p, and miR-548ah-5p was upregulated after treatment, whereas let-7c-5p, let-7f-5p, miR-125a-5p, miR-8a-5p, miR-374b-5p, and miR-98-5p were downregulated (Wielińska et al., 2021). The upregulated and downregulated miRNAs after treatment were found to be associated with tumor suppressor gene TP53, serine/threonine kinase AKT1, proto-oncogene MYC, ubiquitin C, epidermal growth factor receptor, and interleukin-6. Additionally, the upregulated miRNAs were associated with ribosomal protein S27a, MAPK1, ubiquitin B, and VEGFA, while the downregulated miRNAs were associated with ubiquitin A-52 residue ribosomal protein fusion product 1, cyclin D1, tumor suppressor PTEN, and STAT3. The differential expression of exosomal miRNAs observed after 3 months of TNF therapy indicated that exosomes might serve as reliable biomarkers for identifying therapeutic responses, reflecting the reduction of inflammatory responses and control of disease progression by anti-TNF drugs.

Increasing evidence suggests that exosomes play vital roles in disease onset and progression, making them promising therapeutic targets for AS. As natural nanoscale carriers, exosomes not only transferred cargos between cells but also regulated immune pathways (Meng et al., 2020). They could transport functional RNAs, proteins, drugs, or other key metabolic molecules, conferring clinical therapeutic potential. Blocking exosome trafficking and immune pathway modulation could effectively inhibit AS pathogenesis and progression (Tavasolian et al., 2023). Based on the inflammatory response and pathological new bone formation in AS, preventing the release of exosomal inflammatory factors might help better control disease progression (Wang et al., 2024). Exosomes act as transport systems not only for endogenous molecules but also for synthetic drugs, providing a clearer direction for therapeutic research (Liang et al., 2021). Exosomes have been engineered through genetic, physical, and chemical approaches to serve as drug-delivery systems for treating inflammatory bowel disease, such as using CX5461 (an RNA polymerase inhibitor) as an immunosuppressant (Zhang et al., 2024). Although CX5461 could inhibit M1 macrophage proliferation and promote apoptosis to regulate inflammation, its instability under physiological conditions and poor therapeutic effect via injection limited its application. Conversely, sophoridine-derived exosomal nanoparticles demonstrated excellent targeting and stability in inflammatory bowel disease therapy, precisely reaching inflamed colonic sites, representing a novel therapeutic strategy with inflammation-targeting capacity. Wang P et al. showed that exosomes from M1-polarized macrophages could serve as carriers to deliver paclitaxel to tumor tissues, enhancing chemotherapeutic antitumor effects in tumor-bearing mice (Wang et al., 2019), which provided significant inspiration for AS therapy. Zhang Y et al. also demonstrated that exosomes could promote bone tissue repair and regeneration (Zhang Y. et al., 2022), which was highly beneficial for AS disease control.

## 6 Conclusion

AS is a disease with high prevalence in young and middle-aged adults. Advances in molecular biology and the maturation of bioinformatics have facilitated the identification and study of numerous differentially expressed exosomal components associated with AS. As diagnosis currently relies on clinical manifestations and imaging, and lacks ideal blood-based diagnostic markers, exosomes—carrying cargos such as miRNAs, circRNAs, lncRNAs, and proteins—possess functions in immune response and inflammation and may serve as potential biomarkers for early diagnosis of AS. Although the precise mechanisms underlying exosomal circulation, immune regulation, and function in AS remain to be fully elucidated, these issues are expected to be resolved with further research. Exosome-based diagnostic strategies and novel therapeutic approaches may eventually provide clinical benefit for patients with AS, pending further validation.

## References

[B1] Agrawal P. Tote S. Sapkale B. (2024). Diagnosis and treatment of ankylosing spondylitis. Cureus 16, e52559. 10.7759/cureus.52559 38371049 PMC10874590

[B2] Barrett S. P. Salzman J. (2016). Circular RNAs: analysis, expression and potential functions. Development 143, 1838–1847. 10.1242/dev.128074 27246710 PMC4920157

[B3] Bauer K. M. Round J. L. O’Connell R. M. (2022). No small matter: emerging roles for exosomal miRNAs in the immune system. FEBS J. 289, 4021–4037. 10.1111/febs.16052 34087046 PMC9545694

[B4] Blanc L. Vidal M. (2018). New insights into the function of Rab GTPases in the context of exosomal secretion. Small GTPases 9, 95–106. 10.1080/21541248.2016.1264352 28135905 PMC5902209

[B5] Chen L.-L. (2016). The biogenesis and emerging roles of circular RNAs. Nat. Rev. Mol. Cell Biol. 17, 205–211. 10.1038/nrm.2015.32 26908011

[B6] Chen L. Liu Z. (2019). Downregulation of FSTL-1 attenuates the inflammation injury during Streptococcus pneumoniae infection by inhibiting the NLRP3 and TLR4/NF-κB signaling pathway. Mol. Med. Rep. 20, 5345–5352. 10.3892/mmr.2019.10752 31638229

[B7] Chen W. Wang F. Wang J. Chen F. Chen T. (2022). The molecular mechanism of long non-coding RNA MALAT1-Mediated regulation of chondrocyte pyroptosis in ankylosing spondylitis. Mol. Cells 45, 365–375. 10.14348/molcells.2022.2081 35680372 PMC9200665

[B8] Cheng Y. Zhang Z. (2025). Expression and clinical significance of microRNA-138-5p and TGF-β3 in peripheral blood of patients with ankylosing spondylitis. Glob. Spine J. 15, 742–748. 10.1177/21925682231209626 37978926 PMC11881152

[B9] Cheng K.-Y. Liu Y. Han Y.-G. Li J.-K. Jia J.-L. Chen B. (2017). Follistatin-like protein 1 suppressed pro-inflammatory cytokines expression during neuroinflammation induced by lipopolysaccharide. J. Mol. Histol. 48, 63–72. 10.1007/s10735-016-9706-z 27913976

[B10] Cocucci E. Meldolesi J. (2015). Ectosomes and exosomes: shedding the confusion between extracellular vesicles. Trends Cell Biol. 25, 364–372. 10.1016/j.tcb.2015.01.004 25683921

[B11] Colombo M. Raposo G. Théry C. (2014). Biogenesis, secretion, and intercellular interactions of exosomes and other extracellular vesicles. Annu. Rev. Cell Dev. Biol. 30, 255–289. 10.1146/annurev-cellbio-101512-122326 25288114

[B12] Conlon G. A. Murray G. I. (2019). Recent advances in understanding the roles of matrix metalloproteinases in tumour invasion and metastasis. J. Pathol. 247, 629–640. 10.1002/path.5225 30582157

[B13] Crossfield S. S. R. Marzo-Ortega H. Kingsbury S. R. Pujades-Rodriguez M. Conaghan P. G. (2021). Changes in ankylosing spondylitis incidence, prevalence and time to diagnosis over two decades. RMD Open 7, e001888. 10.1136/rmdopen-2021-001888 34887345 PMC8663075

[B14] Ding X. Liu J. Sun Y. Chen X. (2024). Triptolide alleviates the development of inflammation in ankylosing spondylitis *via* the NONHSAT227927.1/JAK2/STAT3 pathway. Exp. Ther. Med. 27, 17. 10.3892/etm.2023.12305 38223328 PMC10785042

[B15] Doyle L. M. Wang M. Z. (2019). Overview of extracellular vesicles, their origin, composition, purpose, and methods for exosome isolation and analysis. Cells 8, 727. 10.3390/cells8070727 31311206 PMC6678302

[B16] Fang Y. Liu J. (2023). Novel regulatory role of non-coding RNAs in ankylosing spondylitis. Front. Immunol. 14, 1131355. 10.3389/fimmu.2023.1131355 36911689 PMC9998703

[B17] FangY. LiuJ. LongY. WenJ. HuangD. XinL. (2022). Knockdown of circular RNA hsa_circ_0003307 inhibits synovial inflammation in ankylosing spondylitis by regulating the PI3K/AKT pathway. Adv. Clin. Exp. Med. 31, 781–788. 10.17219/acem/146830 35275449

[B18] Fotoh D. S. Noreldin R. I. Rizk M. S. Elsabaawy M. M. Esaily H. A. (2020). miRNA-451a and miRNA-125a expression levels in ankylosing spondylitis: impact on disease diagnosis, prognosis, and outcomes. J. Immunol. Res. 2020, 2180913. 10.1155/2020/2180913 33426087 PMC7781682

[B19] Gerami M. H. Khorram R. Rasoolzadegan S. Mardpour S. Nakhaei P. Hashemi S. (2023). Emerging role of mesenchymal stem/stromal cells (MSCs) and MSCs-derived exosomes in bone- and joint-associated musculoskeletal disorders: a new frontier. Eur. J. Med. Res. 28, 86. 10.1186/s40001-023-01034-5 36803566 PMC9939872

[B20] Greening D. W. Simpson R. J. (2018). Understanding extracellular vesicle diversity - current status. Expert Rev. Proteomics 15, 887–910. 10.1080/14789450.2018.1537788 30326765

[B21] Greening D. W. Gopal S. K. Xu R. Simpson R. J. Chen W. (2015). Exosomes and their roles in immune regulation and cancer. Semin. Cell Dev. Biol. 40, 72–81. 10.1016/j.semcdb.2015.02.009 25724562

[B22] Hu L. Guan Z. Tang C. Li G. Wen J. (2022). Exosomes derived from microRNA-21 overexpressed adipose tissue-derived mesenchymal stem cells alleviate spine osteoporosis in ankylosing spondylitis mice. J. Tissue Eng. Regen. Med. 16, 634–642. 10.1002/term.3304 35441454

[B23] Huang Y. Feng F. Huang Q. Zheng S. Huang Z. Deng W. (2020). Proteomic analysis of serum-derived extracellular vesicles in ankylosing spondylitis patients. Int. Immunopharmacol. 87, 106773. 10.1016/j.intimp.2020.106773 32679547

[B24] Jafarpour M. Omidvar M. H. Soltani-Zangbar M. S. Dolati S. Ahmadi M. Jadidi-Niaragh F. (2022). The effects of PBMCs-derived exosomes of ankylosing spondylitis patients on T cell profiles. Gene Rep. 26, 101446. 10.1016/j.genrep.2021.101446

[B25] Jeppesen D. K. Fenix A. M. Franklin J. L. Higginbotham J. N. Zhang Q. Zimmerman L. J. (2019). Reassessment of exosome composition. Cell 177, 428–445.e18. 10.1016/j.cell.2019.02.029 30951670 PMC6664447

[B26] Ji W. Lu Y. Ma Z. Gan K. Liu Y. Cheng Y. (2022). Triptolide attenuates inhibition of ankylosing spondylitis-derived mesenchymal stem cells on the osteoclastogenesis through modulating exosomal transfer of circ-0110634. J. Orthop. Transl. 36, 132–144. 10.1016/j.jot.2022.05.007 36185580 PMC9489540

[B27] Kalluri R. LeBleu V. S. (2020). The biology, function, and biomedical applications of exosomes. Science 367, eaau6977. 10.1126/science.aau6977 32029601 PMC7717626

[B28] Kasper G. Glaeser J. D. Geissler S. Ode A. Tuischer J. Matziolis G. (2007). Matrix metalloprotease activity is an essential link between mechanical stimulus and mesenchymal stem cell behavior. Stem Cells 25, 1985–1994. 10.1634/stemcells.2006-0676 17495113

[B29] Khan M. A. Yong S.-B. Wei J. C.-C. (2023). Ankylosing spondylitis: history, epidemiology, and HLA-B27. Int. J. Rheum. Dis. 26, 413–414. 10.1111/1756-185X.14547 36859756

[B30] Kou J. Bie Y. Liu M. Wang L. Liu X. Sun Y. (2024). Identification and bioinformatics analysis of lncRNAs in serum of patients with ankylosing spondylitis. BMC Musculoskelet. Disord. 25, 291. 10.1186/s12891-024-07396-z 38622662 PMC11017588

[B31] Krylova S. V. Feng D. (2023). The machinery of exosomes: biogenesis, release, and uptake. Int. J. Mol. Sci. 24, 1337. 10.3390/ijms24021337 36674857 PMC9865891

[B32] Kumari S. Lausted C. Scherler K. Ng A. H. C. Lu Y. Lee I. (2024). Approaches and challenges in characterizing the molecular content of extracellular vesicles for biomarker discovery. Biomolecules 14, 1599. 10.3390/biom14121599 39766306 PMC11674167

[B33] Lee H. Groot M. Pinilla-Vera M. Fredenburgh L. E. Jin Y. (2019). Identification of miRNA-rich vesicles in bronchoalveolar lavage fluid: insights into the function and heterogeneity of extracellular vesicles. J. Control Release 294, 43–52. 10.1016/j.jconrel.2018.12.008 30529727 PMC6372374

[B34] Lee H.-I. Park K.-J. Kim H.-J. Choi A.-R. Jin S.-H. Kim T.-J. (2022). Serum miR-3620-3p as a novel biomarker for ankylosing spondylitis. J. Rheum. Dis. 29, 33–39. 10.4078/jrd.2022.29.1.33 37476698 PMC10324918

[B35] Li J. Xie X. Liu W. Gu F. Zhang K. Su Z. (2021). MicroRNAs as biomarkers for the diagnosis of ankylosing spondylitis: a systematic review and meta-analysis. Front. Med. (Lausanne) 8, 701789. 10.3389/fmed.2021.701789 34447765 PMC8383110

[B36] Li H. Wang L. Zhu J. Xiao J. Yang H. Hai H. (2023a). Diagnostic serum biomarkers associated with ankylosing spondylitis. Clin. Exp. Med. 23, 1729–1739. 10.1007/s10238-022-00958-2 36459277

[B37] Li Z. Khan M. K. van der Linden S. M. Winkens B. Villiger P. M. Baumberger H. (2023b). HLA-B27, axial spondyloarthritis and survival. Ann. Rheum. Dis. 82, 1558–1567. 10.1136/ard-2023-224434 37679034

[B38] Liang Y. Duan L. Lu J. Xia J. (2021). Engineering exosomes for targeted drug delivery. Theranostics 11, 3183–3195. 10.7150/thno.52570 33537081 PMC7847680

[B39] Liao H.-T. Tsai C.-Y. (2023). Cytokines and regulatory T cells in ankylosing spondylitis. Bone Jt. Res. 12, 133–137. 10.1302/2046-3758.122.BJR-2022-0195.R1 37051816 PMC10003037

[B40] Liu W. Huang L. Zhang C. Liu Z. (2019). lncRNA MEG3 is downregulated in ankylosing spondylitis and associated with disease activity, hospitalization time and disease duration. Exp. Ther. Med. 17, 291–297. 10.3892/etm.2018.6921 30651794 PMC6307436

[B41] Liu C. Liang T. Zhang Z. Chen J. Xue J. Zhan X. (2022). Transfer of microRNA-22-3p by M2 macrophage-derived extracellular vesicles facilitates the development of ankylosing spondylitis through the PER2-mediated Wnt/β-catenin axis. Cell Death Discov. 8, 269. 10.1038/s41420-022-00900-1 35606376 PMC9126881

[B42] Liu Z. Liu Y. Li Y. Xu S. Wang Y. Zhu Y. (2024). ECM stiffness affects cargo sorting into MSC-EVs to regulate their secretion and uptake behaviors. J. Nanobiotechnology 22, 124. 10.1186/s12951-024-02411-w 38515095 PMC10956366

[B43] Longevity O. M. A. C. (2024). Retracted: exosome: function and application in inflammatory bone diseases. Oxid. Med. Cell Longev. 2024, 9806854. 10.1155/2024/9806854 38234518 PMC10791365

[B44] Lu L. Sun S. Li H. Xie Y. (2023). Functional mechanism of miR-92b-3p in osteogenic differentiation of fibroblasts in patients with ankylosing spondylitis *via* the TOB1/BMP/Smad pathway. J. Orthop. Surg. Res. 18, 402. 10.1186/s13018-023-03850-1 37268992 PMC10236738

[B45] Luo Q. Fu B. Zhang L. Guo Y. Huang Z. Li J. (2020). Expression and clinical significance of circular RNA hsa_circ_0079787 in the peripheral blood of patients with axial spondyloarthritis. Mol. Med. Rep. 22, 4197–4206. 10.3892/mmr.2020.11520 33000244 PMC7533439

[B46] Mahnke J. Schumacher V. Ahrens S. Käding N. Feldhoff L. M. Huber M. (2016). Interferon Regulatory Factor 4 controls TH1 cell effector function and metabolism. Sci. Rep. 6, 35521. 10.1038/srep35521 27762344 PMC5071867

[B47] Meng W. He C. Hao Y. Wang L. Li L. Zhu G. (2020). Prospects and challenges of extracellular vesicle-based drug delivery system: considering cell source. Drug Deliv. 27, 585–598. 10.1080/10717544.2020.1748758 32264719 PMC7178886

[B48] Milane L. Singh A. Mattheolabakis G. Suresh M. Amiji M. M. (2015). Exosome mediated communication within the tumor microenvironment. J. Control Release 219, 278–294. 10.1016/j.jconrel.2015.06.029 26143224

[B49] Moro A. Qin H. Yuan S. Li H. Liao S. Yang J. (2025). Mechanical growth factor inhibited syndesmophyte formation and the progression of osteoarthritis and ankylosing spondylitis-like symptoms in HLA-B27/Hu-β2m transgenic rats. Arthritis Res. Ther. 27, 218. 10.1186/s13075-025-03677-7 41272763 PMC12639975

[B50] Murphy S. N. Nguyen B. A. Singh R. Brown N. J. Shahrestani S. Neal M. T. (2022). A brief human history of ankylosing spondylitis: a scoping review of pathogenesis, diagnosis, and treatment. Surg. Neurol. Int. 13, 297. 10.25259/SNI_294_2022 35928330 PMC9345125

[B51] Navarro-Compán V. Sepriano A. El-Zorkany B. van der Heijde D. (2021). Axial spondyloarthritis. Ann. Rheum. Dis. 80, 1511–1521. 10.1136/annrheumdis-2021-221035 34615639

[B52] Nik Mohamed Kamal N. N. S. B. Shahidan W. N. S. (2019). Non-Exosomal and exosomal circulatory MicroRNAs: which are more valid as biomarkers? Front. Pharmacol. 10, 1500. 10.3389/fphar.2019.01500 32038230 PMC6984169

[B53] Ospelt C. (2024). Annals of the Rheumatic Diseases collection on epigenetics: from three dimensional chromatin organisation to microRNA. Ann. Rheum. Dis. 83, 821–825. 10.1136/ard-2023-224857 38123909

[B54] Pan B. T. Johnstone R. M. (1983). Fate of the transferrin receptor during maturation of sheep reticulocytes *in vitro:* selective externalization of the receptor. Cell 33, 967–978. 10.1016/0092-8674(83)90040-5 6307529

[B55] Pishgahi A. Abolhasan R. Danaii S. Amanifar B. Soltani-Zangbar M. S. Zamani M. (2020). Immunological and oxidative stress biomarkers in Ankylosing Spondylitis patients with or without metabolic syndrome. Cytokine 128, 155002. 10.1016/j.cyto.2020.155002 31986444

[B56] Raposo G. Stahl P. D. (2019). Extracellular vesicles: a new communication paradigm? Nat. Rev. Mol. Cell Biol. 20, 509–510. 10.1038/s41580-019-0158-7 31324871

[B57] Remalante-Rayco P. Nakamura A. (2024). Year in review: novel insights in the pathogenesis of spondyloarthritis - SPARTAN 2024 annual meeting proceedings. Curr. Rheumatol. Rep. 27, 9. 10.1007/s11926-024-01176-3 39731620

[B58] Robinson P. C. van der Linden S. Khan M. A. Taylor W. J. (2021). Axial spondyloarthritis: concept, construct, classification and implications for therapy. Nat. Rev. Rheumatol. 17, 109–118. 10.1038/s41584-020-00552-4 33361770

[B59] Saleem M. Shahzad K. A. Marryum M. Singh S. Zhou Q. Du S. (2024). Exosome-based therapies for inflammatory disorders: a review of recent advances. Stem Cell Res. Ther. 15, 477. 10.1186/s13287-024-04107-2 39695750 PMC11657721

[B60] Sekar D. (2021). Implications of microRNA 21 and its involvement in the treatment of different type of arthritis. Mol. Cell Biochem. 476, 941–947. 10.1007/s11010-020-03960-y 33136235

[B61] Sieper J. Poddubnyy D. (2017). Axial spondyloarthriti. Lancet 390, 73–84. 10.1016/S0140-6736(16)31591-4 28110981

[B62] Skotland T. Sandvig K. Llorente A. (2017). Lipids in exosomes: current knowledge and the way forward. Prog. Lipid Res. 66, 30–41. 10.1016/j.plipres.2017.03.001 28342835

[B63] Sun R. Wang X. Sun X. Zhao B. Zhang X. Gong X. (2022). Emerging roles of long non-coding RNAs in ankylosing spondylitis. Front. Immunol. 13, 790924. 10.3389/fimmu.2022.790924 35222376 PMC8866863

[B64] Sung S.-E. Park W.-T. Choi J.-H. Kim Y.-I. Ma M.-J. Son W.-S. (2025). Identifying novel biomarkers for ankylosing spondylitis through proteomic profiling of serum-derived extracellular vesicles. Clin. Exp. Med. 25, 227. 10.1007/s10238-025-01718-8 40591022 PMC12213980

[B65] Tam L.-S. Gu J. Yu D. (2010). Pathogenesis of ankylosing spondylitis. Nat. Rev. Rheumatol. 6, 399–405. 10.1038/nrrheum.2010.79 20517295

[B66] Tan H. Ren R. Zhang J. Huang Z. Niu Q. Yang B. (2022). Analysis of inflammation-related microRNA expression in patients with ankylosing spondylitis. Immunol. Res. 70, 23–32. 10.1007/s12026-021-09249-6 34743291

[B67] Tauro B. J. Greening D. W. Mathias R. A. Ji H. Mathivanan S. Scott A. M. (2012). Comparison of ultracentrifugation, density gradient separation, and immunoaffinity capture methods for isolating human colon cancer cell line LIM1863-derived exosomes. Methods 56, 293–304. 10.1016/j.ymeth.2012.01.002 22285593

[B68] Tavasolian F. Inman R. D. (2023). Biology and therapeutic potential of mesenchymal stem cell extracellular vesicles in axial spondyloarthritis. Commun. Biol. 6, 413. 10.1038/s42003-023-04743-z 37059822 PMC10104809

[B69] Tavasolian F. Hosseini A. Z. Mirzaei A. Abdollahi E. Jandaghi P. Soudi S. (2020). Unfolded protein response-mediated modulation of mesenchymal stem cells. IUBMB Life 72, 187–197. 10.1002/iub.2154 31444957

[B70] Tavasolian F. Pastrello C. Ahmed Z. Jurisica I. Inman R. D. (2022). Vesicular traffic-mediated cell-to-cell signaling at the immune synapse in Ankylosing Spondylitis. Front. Immunol. 13, 1102405. 10.3389/fimmu.2022.1102405 36741392 PMC9889860

[B71] Tavasolian F. Lively S. Pastrello C. Tang M. Lim M. Pacheco A. (2023). Proteomic and genomic profiling of plasma exosomes from patients with ankylosing spondylitis. Ann. Rheum. Dis. 82, 1429–1443. 10.1136/ard-2022-223791 37532285

[B72] van Niel G. Carter D. R. F. Clayton A. Lambert D. W. Raposo G. Vader P. (2022). Challenges and directions in studying cell-cell communication by extracellular vesicles. Nat. Rev. Mol. Cell Biol. 23, 369–382. 10.1038/s41580-022-00460-3 35260831

[B73] Wang P. Wang H. Huang Q. Peng C. Yao L. Chen H. (2019). Exosomes from M1-Polarized macrophages enhance Paclitaxel antitumor activity by activating macrophages-mediated inflammation. Theranostics 9, 1714–1727. 10.7150/thno.30716 31037133 PMC6485189

[B74] Wang T. Meng S. Chen P. Wei L. Liu C. Tang D. (2021). Comprehensive analysis of differentially expressed mRNA and circRNA in ankylosing spondylitis patients’ platelets. Exp. Cell Res. 409, 112895. 10.1016/j.yexcr.2021.112895 34717918

[B75] Wang K. Lu J. Song C. Qiao M. Li Y. Chang M. (2024). Extracellular vesicles derived from ligament tissue transport Interleukin-17A to mediate ligament-to-bone crosstalk in ankylosing spondylitis. Adv. Sci. (Weinh) 11, e2406876. 10.1002/advs.202406876 39308181 PMC11633500

[B76] Wei H. Chen Q. Lin L. Sha C. Li T. Liu Y. (2021). Regulation of exosome production and cargo sorting. Int. J. Biol. Sci. 17, 163–177. 10.7150/ijbs.53671 33390841 PMC7757038

[B77] Wielińska J. Crossland R. E. Łacina P. Świerkot J. Bugaj B. Dickinson A. M. (2021). Exploring the extracellular vesicle MicroRNA expression repertoire in patients with rheumatoid arthritis and ankylosing Spondylitis treated with TNF inhibitors. Dis. Markers 2021, 2924935. 10.1155/2021/2924935 34691284 PMC8529175

[B78] Wu J. Yan L. Chai K. (2021). Systemic immune-inflammation index is associated with disease activity in patients with ankylosing spondylitis. J. Clin. Lab. Anal. 35, e23964. 10.1002/jcla.23964 34418163 PMC8418483

[B79] Wu L. Zhou L. An J. Shao X. Zhang H. Wang C. (2023). Comprehensive profiling of extracellular vesicles in uveitis and scleritis enables biomarker discovery and mechanism exploration. J. Transl. Med. 21, 388. 10.1186/s12967-023-04228-x 37322475 PMC10273650

[B80] Xiao J. Joseph S. Xia M. Teng F. Chen X. Huang R. (2022). Circular RNAs acting as miRNAs’ sponges and their roles in stem cells. J. Clin. Med. 11, 2909. 10.3390/jcm11102909 35629034 PMC9145679

[B81] Xie Z. Wang P. Li Y. Deng W. Zhang X. Su H. (2016). Imbalance between bone morphogenetic protein 2 and noggin induces abnormal osteogenic differentiation of mesenchymal stem cells in ankylosing spondylitis. Arthritis Rheumatol. 68, 430–440. 10.1002/art.39433 26413886

[B82] Xie F. Liu Y.-L. Chen X.-Y. Li Q. Zhong J. Dai B.-Y. (2020). Role of MicroRNA, LncRNA, and exosomes in the progression of osteoarthritis: a review of recent literature. Orthop. Surg. 12, 708–716. 10.1111/os.12690 32436304 PMC7307224

[B83] Yi L. Song C. Liu Y. Li D. Xiao T. Guo X. (2023). Down-regulation of long noncoding RNA HULC inhibits the inflammatory response in ankylosing spondylitis by reducing miR-556-5p-mediated YAP1 expression. J. Orthop. Surg. Res. 18, 551. 10.1186/s13018-023-04003-0 37525215 PMC10388530

[B84] Yildirim T. Yesilada E. Eren F. Apaydin H. Gulbay G. (2021). Assessment of plasma microRNA potentials as a non-invasive biomarker in patients with axial spondyloarthropathy. Eur. Rev. Med. Pharmacol. Sci. 25, 620–625. 10.26355/eurrev_202101_24620 33577015

[B85] Zarovni N. Corrado A. Guazzi P. Zocco D. Lari E. Radano G. (2015). Integrated isolation and quantitative analysis of exosome shuttled proteins and nucleic acids using immunocapture approaches. Methods 87, 46–58. 10.1016/j.ymeth.2015.05.028 26044649

[B86] Zhang J. Li S. Li L. Li M. Guo C. Yao J. (2015). Exosome and exosomal microRNA: trafficking, sorting, and function. Genomics Proteomics Bioinforma. 13, 17–24. 10.1016/j.gpb.2015.02.001 25724326 PMC4411500

[B87] Zhang H. Wang L. Li C. Yu Y. Yi Y. Wang J. (2019). Exosome-Induced regulation in inflammatory bowel disease. Front. Immunol. 10, 1464. 10.3389/fimmu.2019.01464 31316512 PMC6611439

[B88] Zhang L. Qu L. Zhang Y. Xu Z. Tang H. (2022a). Differential expression of circular RNAs in plasma exosomes from patients with ankylosing spondylitis. Cell Biol. Int. 46, 649–659. 10.1002/cbin.11760 34989461

[B89] Zhang Y. Tu B. Sha Q. Qian J. (2022b). Bone marrow mesenchymal stem cells-derived exosomes suppress miRNA-5189-3p to increase fibroblast-like synoviocyte apoptosis *via* the BATF2/JAK2/STAT3 signaling pathway. Bioengineered 13, 6767–6780. 10.1080/21655979.2022.2045844 35246006 PMC8973596

[B90] Zhang W. Li M. Li X. Wang X. Liu Y. Yang J. (2023). Global trends and research status in ankylosing spondylitis clinical trials: a bibliometric analysis of the last 20 years. Front. Immunol. 14, 1328439. 10.3389/fimmu.2023.1328439 38288126 PMC10823346

[B91] Zhang M. Xu X. Su L. Zeng Y. Lin J. Li W. (2024). Oral administration of Sophora Flavescens-derived exosomes-like nanovesicles carrying CX5461 ameliorates DSS-induced colitis in mice. J. Nanobiotechnology 22, 607. 10.1186/s12951-024-02856-z 39379937 PMC11463058

[B92] Zheng Y. Chaudhry A. Kas A. deRoos P. Kim J. M. Chu T.-T. (2009). Regulatory T-cell suppressor program co-opts transcription factor IRF4 to control T(H)2 responses. Nature 458, 351–356. 10.1038/nature07674 19182775 PMC2864791

[B93] Zhu W. He X. Cheng K. Zhang L. Chen D. Wang X. (2019). Ankylosing spondylitis: etiology, pathogenesis, and treatments. Bone Res. 7, 22. 10.1038/s41413-019-0057-8 31666997 PMC6804882

[B94] Zou Y.-C. Yan L.-M. Gao Y.-P. Wang Z.-Y. Liu G. (2020). miR-21 may Act as a potential mediator between inflammation and abnormal bone formation in ankylosing spondylitis based on TNF-α concentration-dependent manner through the JAK2/STAT3 pathway. Dose Response 18, 1559325819901239. 10.1177/1559325819901239 32009856 PMC6974759

